# Long-Term Endoscopic Follow-Up of Patients with Chronic Radiation Proctopathy after Brachytherapy for Prostate Cancer

**DOI:** 10.1155/2016/1414090

**Published:** 2016-06-09

**Authors:** Masahiro Ohtani, Hiroyuki Suto, Takuto Nosaka, Yasushi Saito, Yoshihiko Ozaki, Ryoko Hayama, Tatsushi Naito, Kazuto Takahashi, Kazuya Ofuji, Hidetaka Matsuda, Katsushi Hiramatsu, Tomoyuki Nemoto, Hiroki Shioura, Hirohiko Kimura, Yoshitaka Aoki, Osamu Yokoyama, Yasunari Nakamoto

**Affiliations:** ^1^Second Department of Internal Medicine, Faculty of Medical Sciences, University of Fukui, 23-3 Matsuokashimoaizuki, Eiheiji-cho, Yoshida-gun, Fukui 910-1193, Japan; ^2^Department of Radiology, Faculty of Medical Sciences, University of Fukui, Fukui 910-1193, Japan; ^3^Department of Urology, Faculty of Medical Sciences, University of Fukui, Fukui 910-1193, Japan

## Abstract

*Background*. Chronic radiation proctopathy (CRP) is late toxicity and associated with morbidity.* Aim*. To investigate the predictors of prognosis in patients with CRP after brachytherapy (BT).* Methods*. One hundred four patients with prostate cancer were treated with BT or BT followed by external-beam radiotherapy (BT + EBRT). We retrospectively investigated the 5-year incidence of rectal bleeding and endoscopic findings of CRP using the Vienna Rectoscopy Score (VRS). Twenty patients with VRS ≥ 1 were divided into the improved VRS group without treatment, unchanged VRS group, and treated group. The parameters associated with alteration of VRS were analyzed.* Results*. The incidence of rectal bleeding was 24%. The risk of rectal bleeding was higher in patients treated with BT + EBRT compared to those treated with BT (*p* < 0.0001). The incidence of superficial microulceration was higher in the improved VRS group than in the unchanged VRS group (*p* < 0.05). The incidence of multiple confluent telangiectasia or superficial ulcers > 1 cm^2^ was higher in the treated group than in both the improved and unchanged VRS groups (*p* < 0.05).* Conclusions*. Patients treated with BT + EBRT have a high risk of CRP. Endoscopic findings were useful for prognostic prediction of CRP.

## 1. Introduction

Prostate cancer is the most commonly diagnosed cancer and the second-leading cause of cancer-related mortality in men over the age of 40 years in the United States [1], with the incidence of prostate cancer in Japan increasing in recent years [2]. For localized prostate cancer, radiation therapy is an effective treatment modality. Since the approval of ^125^I seed source in 2003, ^125^I brachytherapy (BT) has become one of the standard treatment modalities for low- to intermediate-risk prostate cancer in Japan. In addition, a combination of BT and external-beam radiation therapy (EBRT) is used for intermediate- to high-risk prostate cancer.

Chronic radiation proctopathy (CRP) is a late gastrointestinal (GI) toxicity and one of the common adverse effects after radiation therapy. Histopathological features of CRP are ischemic endarteritis and fibrosis in the submucosa [3]. The symptoms of CRP include hematochezia, tenesmus, diarrhea, fecal incontinence, and defecatory urgency. The most common complaint is rectal bleeding. CRP is temporary and self-limiting in approximately 95% of all patients; however, patients with severe recurrent hemorrhage may require hospitalization and blood transfusions [3]. Furthermore, various treatment modalities have been reported for the management of CRP, including endoscopic argon plasma coagulation (APC) [4], dilute formalin topical treatment [5], hyperbaric oxygen (HBO) therapy [6], and sucralfate enema [7]. For the evaluation of CRP, endoscopic examination is the most important modality. On endoscopy, the rectal mucosa may show varying appearances, from telangiectasia (TE) to ulceration (UL). Although the relationship between the symptoms and endoscopic findings of CRP has been investigated, literature about long-term follow-up with endoscopic examination is limited [8]. A study has reported the endoscopic findings at 12 and 65 months after EBRT, showing improvement in TE [8]. However, the long-term endoscopic evaluation of CRP after BT has not been reported. Furthermore, the indicators of medical treatment including APC or HBO therapy for rectal bleeding caused by CRP are unclear. Therefore, the purpose of this study was to evaluate the risk and prognostic factors of rectal bleeding after BT for prostate cancer.

## 2. Patients and Methods

### 2.1. Patients

A retrospective medical chart review was performed on consecutive patients with prostate cancer underwent brachytherapy between May 2006 and September 2009 at University of Fukui Hospital. This study was approved by institutional review board of University of Fukui, with the IRB number of 20150119. The inclusion criteria consist of histological diagnosis of prostate cancer and treatment with BT alone or BT followed by EBRT boost. The exclusion criteria were subjects with incompletion of follow-up after BT and coexistence of prostate and rectal cancer. Patients were implanted with ^125^I seeds (OncoSeed; Nihon Medi-physics Co., Japan) by using modified peripheral loading techniques and a Mick applicator (Mick Radionuclear Instruments, Bronx, NY, USA). The prescribed dose was 144 Gy in patients undergoing BT only and 105 Gy in those undergoing BT + EBRT [9]. For combined therapy, BT was performed first, followed by EBRT 4–8 weeks after BT. EBRT was delivered using 10-MV X-rays in two or four fields, with a daily fraction dose of 1.8 Gy, for 5 days per week, up to total dose of 45–55 Gy.

### 2.2. Evaluation of Late Rectal Toxicity

After radiation therapy, patients were routinely followed up every 3 months to record GI toxicity due to radiotherapy. Rectal bleeding was graded according to the National Cancer Institute Common Terminology Criteria for Adverse Event (CTCAE) version 4.0 [10]. Rectal bleeding was defined as four instances of hematochezia in 4 weeks [11]. No rectal bleeding was defined as CTCAE grade 0 for evaluating the relationship between toxicity grade and endoscopic scoring. We evaluated the incidence of rectal bleeding after BT for 5 years and the factors associated with rectal bleeding, including age, extent of the primary tumor in TMN classification, use of antithrombotic therapy, presence or absence of diabetes mellitus, volume of the prostate gland, presence or absence of EBRT boost, number of seeds, and the use of neoadjuvant androgen depletion therapy (ADT). Tumor staging was performed according to the International Union Against Cancer TMN Classification of Malignant tumors 6th edition [12].

### 2.3. Evaluation of Endoscopic Findings of CRP

Several endoscopists performed colonoscopy or rectosigmoidoscopy by using the PCF-Q260AI (Olympus, Japan) for symptoms of CRP, as part of a health check-up or as an examination for fecal occult blood. The endoscopic findings of rectal mucosa were graded using the Vienna Rectoscopy Score (VRS), as reported by Wachter et al. [13]. In brief, endoscopic findings including TE, congested mucosa, UL, stricture, and necrosis were graded according to the following graduation system:

 TE: grade 0, none; grade 1, single TE; grade 2, multiple nonconfluent TE; and grade 3, multiple confluent TE.
 Congested mucosa: grade 0, none; grade 1, focal reddening of the mucosa combined with an edematous mucosa; grade 2, diffuse but nonconfluent reddening of the mucosa combined with an edematous mucosa; and grade 3, diffuse confluent reddening of the mucosa combined with an edematous mucosa.
 UL: grade 0, none; grade 1, superficial microulceration < 1 cm^2^; grade 2, superficial UL > 1 cm^2^; grade 3, deep UL; and grade 4, fistula and perforation.
 Stricture: grade 0, none; grade 1, more than two-thirds of the regular diameter; grade 2, one-third to two-thirds of the regular diameter; grade 3, less than one-third of the regular diameter; and grade 4, complete obstruction.
 Necrosis: grade 0, none, and grade 1, necrosis.

Each of the five endoscopic parameters were summarized as VRS, from 0 to 5.

The relationship between the VRS and CTCAE grade was analyzed. Furthermore, the 5-year follow-up period was divided into the early period (up to the first 150 weeks after BT) and the late period of posttreatment (more than 150 weeks after BT and thereafter). Patients who had undergone endoscopy in both early and late period were classified into three groups based on their findings, the improved VRS group, the unchanged VRS group, or the treated group (treated with APC or HBO therapy), to evaluate changes of endoscopic findings and to determine their clinical characteristics.

### 2.4. Statistical Analysis

The distribution of clinical parameters for rectal bleeding and endoscopic parameters associated with rectal mucosal alteration was analyzed using the Student *t*-test and Fisher's exact test. The cumulative incidence of rectal bleeding was evaluated with the Kaplan-Meier method, and significant differences between treatment modalities were calculated by the log-rank test. The relationship between VRS and the CTCAE grade, obtained at the time of endoscopic examination, was analyzed by using Spearman's correlation test. All statistical analyses were performed using GraphPad Prism 6 (GraphPad Software, Inc., La Jolla, CA). Statistical significance was defined as *p* < 0.05.

## 3. Results

### 3.1. Patient Characteristics

A total of 104 patients with localized prostate cancer underwent transperineal ^125^I prostate BT between May 2006 and September 2009 at our institute. The characteristics of these patients are shown in Table 1. The mean age was 68.1 years (range: 54–82 years). According to TMN classification, 81 and 23 patients were classified as having T1 and T2 disease, respectively. Twenty-one patients (20.2%) received antithrombotic drugs for coronary artery diseases or cerebral infarction. A total of 12 patients (11.5%) were treated for diabetes mellitus. The mean prostate volume before RT estimated on ultrasonography was 25.6 mL. Combined therapy with EBRT was performed in 53 (51.0%) patients. The median dose of EBRT was 45 Gy (range: 43–55.6 Gy) and median number of ^125^I seeds was 70 (range: 40–100). Thirty-nine patients were treated with neoadjuvant ADT consisting of luteinizing hormone-releasing hormone agonists and antiandrogen.

### 3.2. Risk Factors of Rectal Bleeding after Radiotherapy for Patients with Prostate Cancer

**Figure 1:**
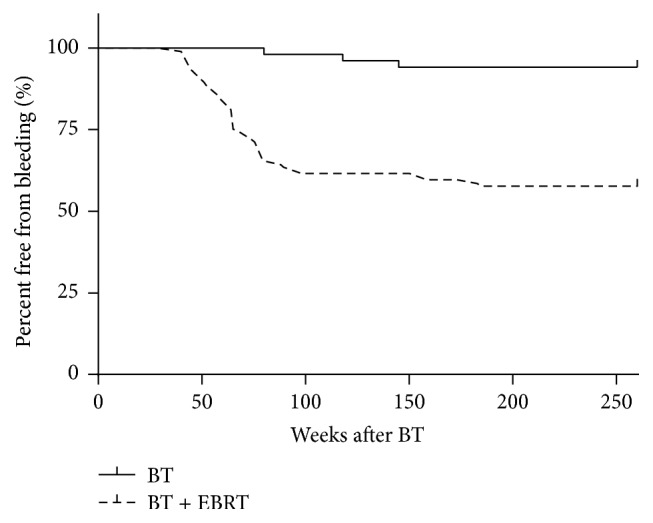
Kaplan-Meier curve for grade ≥ 1 rectal bleeding over 5 years (*p* < 0.0001). BT: brachytherapy; EBRT: external-beam radiotherapy.

Rectal bleeding was observed in 25 patients (24%), with grade 1 (12.5%) being the most common, followed by grade 2 (4.8%) and grade 3 (6.7%) (Table 2). Endoscopy was performed in 56 (53.8%) patients totally and in 24 of 25 patients who experienced rectal bleeding symptoms of CTCAE grade ≥ 1. Iron supplementation was prescribed for 9 patients with anemia due to rectal bleeding. Endoscopic APC and HBO therapy were administered to 5 and 2 patients, respectively (Table 2). All patients who received endoscopic APC or HBO therapy showed improvement in their rectal bleeding symptoms. The occurrence of rectal bleeding was 41.5% in the BT + EBRT group while it was 5.9% in the BT group, indicating a significant association of the presence or absence of EBRT with rectal bleeding of CTCAE grade ≥ 1 (*p* < 0.0001) (Table 3). No significant differences were observed in other factors. In a log-rank test based on the presence or absence of EBRT, a significantly higher occurrence of rectal bleeding was found in the BT + EBRT group compared to the BT group (*p* < 0.0001) (Figure 1), and the Kaplan-Meier curve showed that the first rectal bleeding occurred before the 150th week after BT in the BT group. Conversely, in the BT + EBRT group, rectal bleeding occurred more frequently in the earlier period after BT (50th to 100th week) compared to the BT group. These results suggest that EBRT boost after BT increased the risk of rectal bleeding.

### 3.3. Relationship between Endoscopic Findings and Rectal Bleeding Symptoms and Changes in Endoscopic Findings over Time

**Figure 2:**
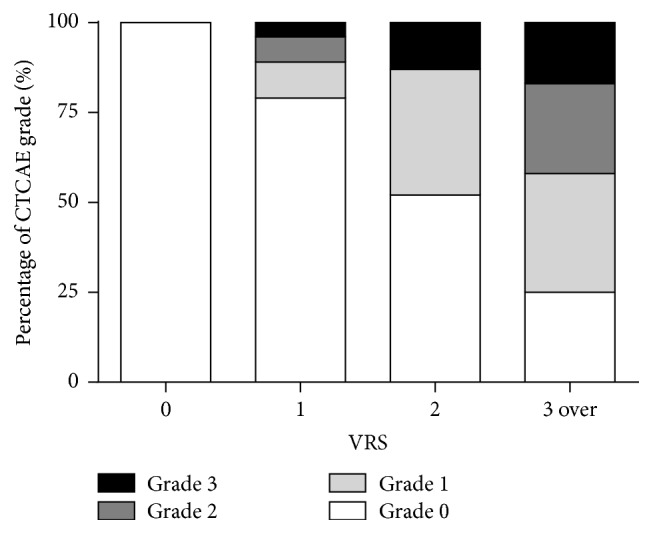
Correlation between VRS and CTCAE grade of rectal bleeding. The statistical significance was analyzed by Spearman's correlation test (*p* < 0.01). VRS: Vienna Rectoscopy Score.

**Figure 3:**
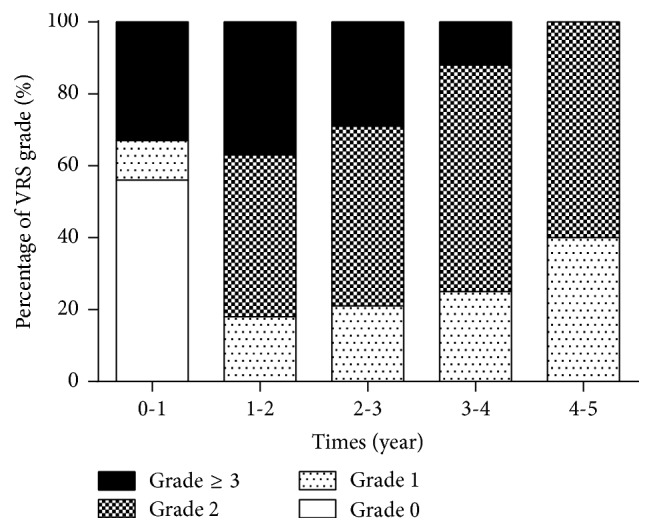
Change in VRS over time, in patients with CTCAE grade ≥ 1. VRS: Vienna Rectoscopy Score.

**Figure 4:**
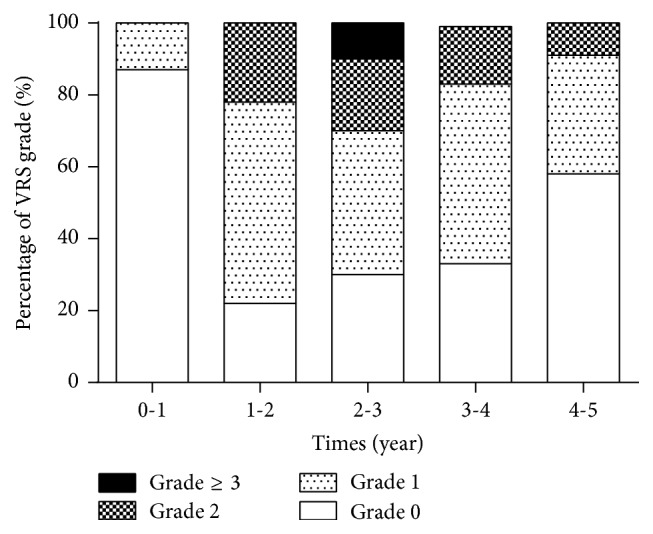
Change in VRS over time, in patients with CTCAE grade 0. VRS: Vienna Rectoscopy Score.

A total of 101 endoscopies were performed in the investigated patients during 5 years (Table 2). The VRS and CTCAE grade in patients who underwent endoscopies were significantly correlated (*p* < 0.01); CTCAE grade ≥ 1 rectal bleeding was observed in 20.7%, 43.7%, and 84.6% when VRS grade was 1, 2, and 3, respectively (Figure 2). A total of 52 endoscopies were performed in patients with CTCAE grade ≥ 1. VRS 0 (normal) was found in 55.6% of the patients who underwent endoscopy within the first year after BT (0 to 1 year) (Figure 3). VRS 1 or higher was found in all patients who underwent endoscopy between 1 and 2 years after BT. Even though 7 patients who received APC or HBO therapy were included, the percentage of patients with VRS 1 increased with time (5 years) while the percentage of patients with VRS ≥ 3 decreased (Figure 3). In patients with CTCAE grade 0, 49 endoscopies were performed. The evaluation of VRS over time in patients without rectal bleeding revealed that VRS 0 was found in 88.9% of patients who underwent endoscopy between 0 years and 1 year after BT (Figure 4). In contrast, VRS ≥ 1 was found in 80% of patients who underwent endoscopy between 0 years and 1 year after BT (Figure 4). With time, the percentage of patients with VRS 0 increased while the percentage of patients with VRS ≥ 2 decreased in patients with CTCAE grade 0. These results indicated that endoscopic findings were correlated with rectal bleeding symptoms, and abnormal findings that were frequently observed 1 year or longer after BT improved over time.

### 3.4. Prognostic Factors of CRP Based on Endoscopic Findings after Treatment

The changes in endoscopic findings in the early and late periods were evaluated in 26 patients. Six patients who underwent endoscopy in the early period after BT had VRS 0, and these patients did not show any change in the endoscopic findings and bleeding symptoms in the late period.

Of 20 patients with VRS ≥ 1 in the early period, 4, 5, and 11 patients were from the treated group with APC or HBO therapy, the improved VRS group without treatment, and the unchanged VRS group, respectively (Table 4). All patients with VRS 1 in the early period also did not show any change of VRS. However, improvement of VRS was observed in 2 of 10 patients with VRS 2 and 3 of 6 patients with VRS ≥ 3. Rectal bleeding symptoms improved over the 5-year course in all patients of both the improved VRS and unchanged VRS groups. The percentage of patients with UL grade 1 was significantly higher in the improved VRS group (60%) than in the unchanged VRS group (0%) (*p* < 0.05) (Table 4). When patients had TE grade 3 and UL grade ≥ 2 along with higher endoscopic severity, 75% of patients in the treated group met the criteria while none satisfied the criteria in the improved VRS group and the unchanged VRS group (*p* < 0.05) (Table 4). No differences were observed among the three groups considering the age; extent of the primary tumor (T); use of neoadjuvant ADT, antithrombotic therapy; volume of the prostate gland; and the presence or absence of EBRT. These results indicated that natural recovery of superficial ulceration (UL grade 1) could be expected in patients with VRS ≥ 2.

## 4. Discussion

In this study, we examined risk factors of rectal bleeding and the prognosis of CRP after BT for prostate cancer. The risk of rectal bleeding significantly increased in the patients treated with BT + EBRT compared to those treated with BT alone. Most episodes of rectal bleeding were observed 1 year or more after BT, and endoscopic findings were correlated with the rectal bleeding score. Overtime evaluation of endoscopic findings after radiotherapy for 5 years revealed that spontaneous recovery was promising if UL grade was 1 or less in the first 150 weeks after BT.

Although the efficacy of radiotherapy for prostate cancer has been recognized, various late toxicities, including rectal bleeding, hematuria, and erectile dysfunction, have been reported, because of the rectum or the urethra adjacent to the prostate gland [14]. The occurrence of rectal bleeding after BT and BT + EBRT is 9.1–27.0% and 18.3–47%, respectively [15–18]. Although BT + EBRT was reported to increase rectal bleeding risk compared to BT [14, 15], no difference in the incidence of toxicity between BT and BT + EBRT has been indicated [11, 18]. Therefore, the rectal bleeding risk and toxicity of these modalities are still controversial. Rectal bleeding risk increased in patients with coronary artery disease while ADT decreased the risk [11]. However, in the present study, no difference was observed in the use of antithrombotic therapy. The use of antithrombotic therapy also results in a rectal bleeding risk after BT or EBRT monotherapy [16, 19]; hence, the limited number of patients in the present study was a possible reason for this difference in results compared to previous studies. In addition, ADT was performed as neoadjuvant therapy and it did not affect rectal bleeding.

As VRS proposed by Wachter et al. varies in grade by TE, congested mucosa, UL, stricture, and necrosis, it is useful for the comprehensive evaluation of endoscopic findings of CRP [13]. VRS has been correlated with rectal GI toxicity caused by pelvic radiotherapy [20, 21]. However, VRS is more sensitive compared to rectal bleeding score systems evaluated by clinical symptoms [13, 21]. A similar result was obtained in our study; no bleeding symptom was observed in 56.3% and 23.1% of patients with VRS 2 and VRS ≥ 3, respectively.

In the present study, severity of endoscopic findings increased during 1 to 2 year(s) after treatment and improved over the 5 years of observation. There are only a limited number of reports about the long-term endoscopic evaluation of CRP. Goldner et al. performed proctosigmoidoscopy at 12 and 24 months in patients with prostate cancer who underwent EBRT (range 70 to 75 Gy) and reported that VRS ≥ 1 was found in 64% and 62% of the patients at 12 and 24 months, respectively [21]. On the other hand, O'Brien et al. performed proctosigmoidoscopy in prostate cancer patients who underwent EBRT with 65 Gy and showed that the incidence of multiple TE in the rectum at 24 months was higher than that at 12 months and that it was lower at 36 months [22]. Furthermore, Goldner et al. performed a long-term follow-up with endoscopy at 12, 24, and 65 months and showed that the percentage of patients with VRS 1 increased and the percentage of patients with VRS 2 decreased with time [8]. The improving tendency of VRS over the course of a long-term follow-up was similar to the results of our study. In this study, no abnormal endoscopic finding was observed in 50% of the patients who presented with rectal bleeding of grade ≥ 1 until the first year after BT. Increased severity of endoscopic findings was observed more often between 1 year and 2 years after BT. Delayed development of rectal mucosal change was observed compared to previous report [21]. This difference can be explained by the fact that EBRT + BT and BT were compared in our study, whereas previous studies evaluated the effects of EBRT alone; moreover, in our study, EBRT boost was initiated at the 4–8th week after BT.

Ippolito et al. performed endoscopy in prostate cancer patients treated with intensity-modulated radiation therapy or three-dimensional conformal radiation therapy, 1 year after treatment, and reported that the occurrence of grade ≥ 2 rectal bleeding was 32% and 47% in patients with VRS ≥ 2 and VRS ≥ 3, respectively, indicating the effectiveness of VRS in predicting rectal bleeding [23]. In the present study, as shown in the Kaplan-Meier curve, most rectal bleeding symptoms occurred between the 50th and 150th week. Hence, we examined whether it would be possible to determine the prognosis of CRP based on the endoscopic findings observed during this period. Our results showed the natural decrease of VRS over time in 31% (5/16) of patients with VRS ≥ 2, as well as possible UL 1 improvement, while mild endoscopic findings (VRS 0-1) remained unchanged. No study report has examined factors affecting the recovery of endoscopic findings. Nevertheless, because the number of patients was limited in this study, more patients will be necessary for further investigation in the future. Although the effectiveness and safety of APC therapy have been demonstrated for rectal bleeding due to CRP [4, 24], patients using nonsteroidal anti-inflammatory drugs have shown the development of stenosis after treatment [25] as well as the occurrence of ulcer [26]. Therefore, the endoscopic findings at the occurrence of symptoms are deemed useful for considering treatment indications such as APC or HBO therapy.

## 5. Conclusion

While the incidence of rectal bleeding is low after BT for prostate cancer, EBRT boost increases its risk. Endoscopy was correlated with bleeding symptoms such as GI toxicity, and it allows an accurate evaluation of rectal mucosal damage. Endoscopy in the first 150 weeks after BT is useful for estimating the long-term prognosis of CRP.

## Figures and Tables

**Table 1:** Patient characteristics.

Characteristic	
Number of patients	104
Age, mean ± SD	68.1 ± 6.6
Clinical stage, *n* (%)	
T1c	81 (77.8)
T2a	16 (15.4)
T2b	4 (3.8)
T2c	3 (2.9)
Use of antithrombotic drugs (%)	21 (20.2)
Presence of DM (%)	12 (11.5)
Volume of prostate, (cc) mean ± SD	25.6 ± 8.3
Treatment	
EBRT boost (%)	53 (51.0)
Median dose of EBRT (range)	45 (43–55.6)
Median number of seeds (range)	70 (40–100)
ADT (%)	39 (37.5)

DM, diabetes mellitus; EBRT, external-beam radiotherapy; ADT, androgen deprivation therapy.

**Table 2:** Incidence of rectal bleeding, endoscopic examination, and treatment.

	*n* (%)
CTCAE rectal bleeding scale	
Grade 1	13 (12.5%)
Grade 2	5 (4.8%)
Grade 3	7 (6.7%)
Total	25 (24.0%)
Endoscopic examination	
Number of patients who underwent endoscopy	56 (53.8%)
Total number of endoscopy	101
Number of patients who underwent endoscopy in group of grade ≥ 1 rectal bleeding	24
Treatment	
Iron supplementation	9
Endoscopic argon plasma coagulation	5
Hyperbaric oxygen therapy	2

**Table 3:** Clinical parameters associated with rectal bleeding.

Parameter	Grade ≥ 1 rectal bleeding (%)	*p *value
Age, years		
≥70	14/43 (32.5)	0.11
<70	11/61 (18.0)	
Stage		
T1	19/81 (23.4)	0.79
T2	6/23 (26.1)	
Use of antithrombotic drugs		
Yes	7/21 (33.3)	0.27
No	18/83 (21.6)	
Presence of DM		
Yes	2/12 (16.6)	0.73
No	23/92 (25.0)	
Prostate volume, cc		
≥25	11/49 (22.4)	0.82
<25	14/55 (25.4)	
EBRT boost		
Yes	22/53 (41.5)	<0.0001
No	3/51 (5.9)	
Number of seeds		
≥70	10/52 (19.2)	0.35
<70	15/52 (28.8)	
Use of ADT		
Yes	8/39 (21.0)	0.64
No	17/65 (10.8)	

DM, diabetes mellitus; EBRT, external-beam radiotherapy; ADT, androgen deprivation therapy.

**Table 4:** Endoscopic and clinical factors for improvement of VRS in patients with VRS ≥ 1 who underwent follow-up endoscopic examination.

	Treated group (HBO or APC)	Improved group	Unchanged group	*p *value
*N*	4	5	11	

VRS				
1	0	0	4	
2	1	2	7	
≥3	3	3	0	

TE score				
0	0	0	0	
1	0	0	4	
2	1	5	7	
3	3	0	0	
UL score				
0	0	2	11	
1	1	3	0	
2	0	0	0	
3	1	0	0	
UL 1 (+)	1/4	3/5^a^	0/7^a^	<0.05^a^
TE 3 or UL ≥ 2	3/4^b^	0/5^b^	0/5^b^	<0.05^b^

Age, years	71.8 ± 8.3	67.2 ± 3.1	69.7 ± 4.2	NS
Stage T1/T2	2/2	3/2	6/1	NS
Use of ADT	2	2	5	NS
Use of antithrombotic drugs	2	1	1	NS
*p* volume, mL	22.5 ± 5.5	25.1 ± 7.0	24.6 ± 9.4	NS
Use of EBRT boost	4	5	7	NS
Number of seeds	71.3 ± 6.3	61.0 ± 13.8	62.9 ± 13.3	NS

VRS, Vienna Rectoscopy Score; HBO, hyperbaric oxygen therapy; APC, argon plasma coagulation; TE, telangiectasia; UL, ulceration; ADT, androgen deprivation therapy; EBRT, external-beam radiotherapy; NS, not significant.

^a^The improved group versus the unchanged group, *p* < 0.05.

^b^The treated group versus the improved group; the treated group versus the unchanged group, *p* < 0.05.
